# PPARγ agonists promote differentiation of cancer stem cells by restraining YAP transcriptional activity

**DOI:** 10.18632/oncotarget.11273

**Published:** 2016-08-12

**Authors:** Upal Basu-Roy, Eugenia Han, Kirk Rattanakorn, Abhilash Gadi, Narendra Verma, Giulia Maurizi, Preethi H. Gunaratne, Cristian Coarfa, Oran D. Kennedy, Michael J. Garabedian, Claudio Basilico, Alka Mansukhani

**Affiliations:** ^1^ Department of Microbiology, NYU School of Medicine, New York, NY, USA; ^2^ Perlmutter Cancer Center, Langone Medical Center, New York, NY, USA; ^3^ Department of Biology and Biochemistry, University of Houston, Houston, Texas, USA; ^4^ Department of Molecular and Cellular Biology, Baylor College of Medicine, Houston, Texas, USA; ^5^ Department of Orthopaedic Surgery, NYU School of Medicine, New York, NY, USA

**Keywords:** osteosarcoma, thiazolidinediones, cancer stem cells, osteoblast lineage, adipocyte

## Abstract

Osteosarcoma (OS) is a highly aggressive pediatric bone cancer in which most tumor cells remain immature and fail to differentiate into bone-forming osteoblasts. However, OS cells readily respond to adipogenic stimuli suggesting they retain mesenchymal stem cell-like properties. Here we demonstrate that nuclear receptor PPARγ agonists such as the anti-diabetic, thiazolidinedione (TZD) drugs induce growth arrest and cause adipogenic differentiation in human, mouse and canine OS cells as well as in tumors in mice. Gene expression analysis reveals that TZDs induce lipid metabolism pathways while suppressing targets of the Hippo-YAP pathway, Wnt signaling and cancer-related proliferation pathways. Significantly, TZD action appears to be restricted to the high Sox2 expressing cancer stem cell population and is dependent on PPARγ expression. TZDs also affect growth and cell fate by causing the cytoplasmic sequestration of the transcription factors SOX2 and YAP that are required for tumorigenicity. Finally, we identify a TZD-regulated gene signature based on Wnt/Hippo target genes and PPARγ that predicts patient outcomes. Together, this work highlights a novel connection between PPARγ agonist in inducing adipogenesis and mimicking the tumor suppressive hippo pathway. It also illustrates the potential of drug repurposing for TZD-based differentiation therapy for osteosarcoma.

## INTRODUCTION

Osteosarcoma is the most common type of primary bone cancer and is a major cause of cancer-related deaths in children and adolescents. The disease is often advanced at presentation, and despite improvements in chemotherapy and surgery over the last few decades, the overall survival rate for metastatic osteosarcoma is only about 30% [1–3]. The prognosis for metastatic and relapsed disease has remained poor for decades and alternate approaches for treatment are needed [3, 4]. The incidence of osteosarcoma is 10-times higher in dogs than in humans and amputation followed by chemotherapy, the standard of care, provides approximately one-year of overall survival [5].

Osteosarcomas are frequent tumor in patients with hereditary retinoblastoma (Rb mutations) and with Li-Fraumeni syndrome (p53 mutations) [6]. Spontaneous osteosarcomas originate at high frequency in mice with a knock-out (KO) of the Rb-1 and p53 genes in the osteoblastic lineage and mimic human disease [7, 8]. Murine and human osteosarcoma contain multipotent cancer stem cells (CSCs) that grow in anchorage-independent conditions as osteospheres, possess tumor-initiating properties, and exhibit enhanced resistance to chemotherapeutic drugs [8–10]. The stem cell transcription factor, Sox2, is overexpressed in several mouse and human osteosarcoma cells as well as patient tumor samples, and its expression portends poor survival in patients [11]. Sox2 also plays a significant role in maintaining CSCs in other tumors [12–14]. In osteosarcoma, we showed that high Sox2 expression marks and maintains tumor-initiating CSCs [15]. Knockdown of Sox2 reduced their transformed properties as well as their ability to form tumors [15]. Sox2 depleted cells exhibit increased Hippo signaling, a tumor suppressive pathway that restrains YAP function and that is inactivated in several cancers [16]. These cells show decreased YAP expression with a concurrent increase in the Hippo pathway activators, Nf2 and WWC1. Thus, our previous work demonstrates that Sox2 antagonizes Hippo signaling in osteosarcoma [17]. YAP is also required for tumorigenicity in OS cells and its knockdown mimics that of Sox2 [17].

Osteosarcoma is thought to arise from mesenchymal lineage stem cells or osteoprogenitors and is considered a disease of defective differentiation in which the cells are blocked in their ability to form mature, bone-forming osteoblasts [7]. As the lack of terminal differentiation is associated with high cell proliferation, driving differentiation and subsequently inhibiting tumor growth presents a potential therapeutic strategy for osteosarcoma. This differentiation therapy (DT) is free of the toxicities associated with chemotherapy and circumvents the chemoresistance issues that often arise in standard therapy [18]. Unlike traditional chemotherapy that targets all proliferating cells, DT is restricted to only those cells that respond to the differentiation-inducing stimulus. DT has been implemented for various cancers notably retinoids for acute promyelocytic leukemia [18].

Though spontaneous primary murine osteosarcoma cell lines are unable to differentiate into mature osteoblasts, they retain the capacity to differentiate into adipocytes [15]. This high adipogenic potential is restricted to the high Sca-1/ high Sox2 expressing CSC population [15]. This led us to hypothesize that adipogenesis may be induced in osteosarcomas via targeted stimulation of peroxisome proliferator-activated receptor gamma (PPARγ), a nuclear receptor that activates genes essential for fat formation [19, 20]. Thiazolidinediones (TZDs) are a class of small-molecule activators of PPARγ. They act as insulin sensitizers, and several TZDs, such as pioglitazone (Pio) and rosiglitazone (Rosi), are used in the treatment of T2 diabetes mellitus. Binding of TZDs to PPARγ leads to heterodimerization with RXR, akin to the activity of endogenous ligands. [19, 21, 22]. It has also been reported that TZDs have anti-cancer effects in lung, colon, and breast cancers [22, 23]. Given the mesenchymal origin of osteosarcoma cells and their ability to respond to adipogenic stimuli, we assessed the potential of TZDs in DT of osteosarcoma as they can be easily transitioned to the clinic through drug repurposing.

In this study we demonstrate that TZDs can inhibit growth and migration of human, mouse and dog OS cells, and induce their adipogenic differentiation. RNA-SEQ analysis reveals that the TZD rosiglitazone induces PPARγ targets and lipid metabolism genes in osteosarcoma cells, while reducing the expression of several cancer-related genes. Interestingly, genes which are targets of YAP are decreased in TZD-treated cells with a concurrent decrease in the nuclear localization of Sox2 and YAP, suggesting that TZD treatment in osteosarcomas restores the effect of tumor suppressive Hippo signaling. We demonstrate that TZDs target the CSC population that express high PPARγ compared to the non-CSC population. TZDs can also cooperate with pharmacological YAP inhibition to inhibit osteosarcoma cell growth. TZD treatment of mice implanted with osteosarcoma cells resulted in reduced growth and increased adipogenesis in the tumors *in vivo* and improved surrounding bone quality around intrafemoral tumors. These studies provide proof of principle that TZDs could have a role as an adjuvant differentiation-inducing therapy in combination with chemotherapeutic agents in the management of osteosarcoma.

## RESULTS

### TZDs inhibit growth and migration and induce adipogenesis of osteosarcoma cells

**Figure 1:**
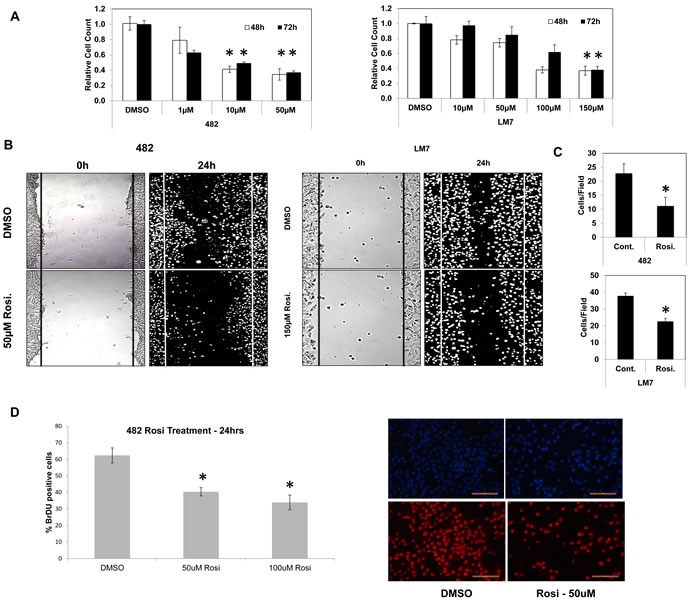
TZD treatment decreases cell proliferation and migration in osteosarcoma cells **A.** Growth of mOS-482 (mouse) and LM7 (human) cells after treatment with control (DMSO), or increasing concentrations of Rosiglitazone at 48- and 72-hours. **B.** Migration scratch assay in mOS-482 cells and LM7 cells, treated for 24 hours with DMSO and Rosiglitazone (mOS-482: 50uM; LM7: 150uM). Photomicrographs of scratch wounds in cell layers shown at time-point 0 hours and 24 hours. **C.** Quantitation of migrating cells counted within the scratch gap averaged over five fields. **D.** Proliferation assay: mOS-482 cells were treated with Rosiglitazone (50 and 100 uM) and DNA synthesis was measured by BrdU incorporation. A representative image of DAPI (top) and BrdU-positive (bottom) cells; magnification = 20X; bar - 200 microns * = *p* < 0.05

Osteosarcomas contain undifferentiated tumor initiating cells or CSCs that express high levels of Sox2 are more efficient at inducing tumor formation and are believed to be responsible for relapse and reseeding of the disease [24]. We reasoned that TZDs may act on this population and stimulate differentiation thereby inhibiting cell growth. To test this, mouse and human osteosarcoma cell lines were treated over a time course with rosiglitazone (Rosi), a PPARγ agonist and analyzed for growth. The murine osteosarcoma cell line mOS-482 and human cells Saos2-LM7 exhibited a concentration-dependent decrease in cell number at 48 and 72 hours of treatment (Figure 1A). Growth arrest was also seen in the human osteosarcoma cell lines OS187 (not shown) and with another TZD, pioglitazone (Pio) (SI1).

The ability of cancer cells to migrate is highly correlated with their tumorigenicity and metastatic potential. To assess the effects of TZDs on osteosarcoma cell migration, an *in vitro* scratch assay was used to monitor the migration of Rosi or DMSO-treated cells across a gap wound made in the cell monolayer. Rosi treatment significantly decreased the migration of mOS-482 and LM7 cells (Figure 1B, 1C). Thus, in addition to growth arrest, the TZDs also inhibit cell migration.

Rosi treated cells also showed a decrease in DNA synthesis measured by BrdU incorporation (Figure 1D). There was no detectable change in apoptosis assessed by TUNEL assay between the control and treated mouse or human cells, suggesting the TZD-induced growth arrest is primarily due to a decrease in proliferation (SI2).

**Figure 2:**
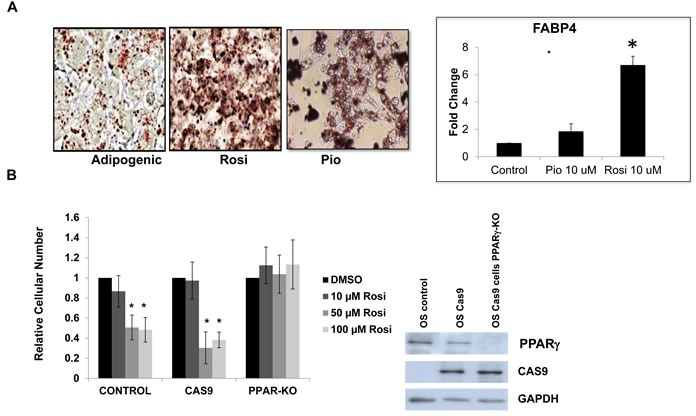
TZD treatment induces adipogenesis in osteosarcoma cells in part through PPARγ activation **A.** Oil Red-O lipid stain of mOS-cells grown in adipogenic media or Rosiglitazone (Rosi) 10uM or Pioglitazone (Pio) 10 uM for 3 days. Mag 40X. Right panel - Relative fold change in mRNA expression of FABP4 measured by qRT-PCR relative to actin as a control. **B.** mOS control, Cas9-expressing or Cas9-PPARγ knockout cells were treated with increasing concentrations of Rosi, as indicated and cell number was determined after 48 hours. Right Panel - Western blot confirming PPARγ deletion in mOS cells expressing PPARγ specific guide RNA.

We had previously demonstrated that OS cells are impaired in their ability to undergo osteogenic differentiation, but paradoxically still retain the ability to undergo adipogenesis [15]. While it is known that TZDs influence adipose-lineage cells and regulate adipose tissue, their effect on adipogenesis in osteosarcoma cells has not been explored [25, 26] We examined whether TZDs Rosi and Pio induced adipogenesis in mouse OS cells. Figure 2A shows that compared to adipogenic media alone, Rosi or Pio treated OS cells undergo enhanced adipogenic differentiation as assessed by an increase in intracellular lipids stained with Oil-Red-O. Increased adipogenesis was confirmed by measuring the expression of the adipocyte-marker genes FABP4 (Figure 2A). This enhanced adipogenesis was also seen in human LM7 cells (SI3). Thus, *in vitro* treatment of mouse and human osteosarcoma cells with the TZDs inhibits cell proliferation and migration, while stimulating adipogenic differentiation.

Canine osteosarcoma shares many similarities with the human disease, including extreme genetic heterogeneity [5, 27]. Canine osteosarcomas also contain a putative CSC population that expresses high levels of Sox2. To determine whether dog OS cells responded to TZDs as the human and mouse cells, OSA2, a spontaneous dog osteosarcoma cell line was treated with Rosi. OSA2 cells also exhibited concentration dependent decrease in growth (SI4). Adipogenic differentiation with Rosi also showed increased Oil-Red-O stain in Rosi-treated dog cells (SI4), confirming that the anti-proliferative and pro-differentiating effects of TZDs is applicable to osteosarcoma across multiple species.

The endocrine factor fibroblast growth factor 21 (FGF21) is being explored as a potential treatment for obesity and diabetes as it enhances insulin sensitivity and decreases triglyceride levels [28, 29]. Mechanistically, this endocrine factor activates the MAP kinase cascade by binding to an FGFR along with the cofactor β-Klotho [30]. FGF21 stimulates adipogenesis in bone marrow mesenchymal stem cells via potentiating the effects of PPARγ [28]. Though both FGF21 and TZDs have been found to enhance adipogenesis in mesenchymal stem cells [28], their cooperative effects on the differentiation process in osteosarcoma cells have not been elucidated. We found that co-treatment of mOS-482 cells with TZDs Rosi or Pio and FGF21 showed increased adipogenesis and enhanced expression of adipocyte-specific gene (FABP4) compared to the TZDs alone (SI5). Similar results were seen with human osteosarcoma cells Saos2-LM7 and OS187 cells. These findings implicate PPARγ induction by TZD and FGF21 treatment as a strong promoter of adipogenesis in osteosarcoma cells and support the finding that activation of this nuclear receptor is sufficient for adipogenesis. [20, 21].

### TZDs action requires PPARγ in osteosarcoma cells

TZDs can have effects on cell physiology that are unrelated to their function as PPARγ [31]. To better pinpoint the mechanisms through which TZDs affect the phenotype of OS cells, we knocked out the PPARγ gene in mouse OS cells using CRISPR/CAS technology and determined the response of the PPARγ knockout (KO) cells to TZDs. The results shown in Figure 2B indicate that cells expressing CAS9 and a guide RNA targeting PPARγ are resistant to the growth inhibitory effects of TZDs (Figure 2B) as well as to adipogenic differentiation. This resistance is not seen in control cells expressing cas9 alone. Sequencing of the PPARγ in isolated clones exhibiting resistance to TZDs revealed deletions starting in exon 3 where the guide RNA is targeted in the PPARγ gene (SI6). Thus these results demonstrate that the TZD effects on osteosarcoma cells are mediated by PPARγ activation and not by unrelated or off-target mechanisms.

### TZDs target osteosarcoma cancer stem cells

We have previously shown that in murine osteosarcoma cell lines, Sox2 nuclear expression correlates with expression of the cell surface antigen Sca-1 [15]. The Sca-1Hi fraction has a higher propensity to form adipocytes and is impaired in osteogenic differentiation, compared to their Sca-1 Lo counterparts [15]. We showed that high tumor forming capacity resided in the Sca-1-Hi cells which comprise the cancer stem cell population [17].

**Figure 3:**
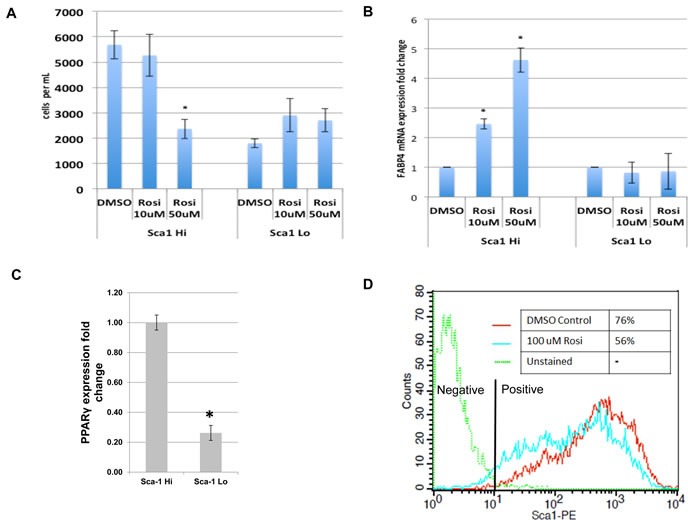
TZDs target the cancer stem cell population of osteosarcomas mOS-482 cells were fractionated into Sca-1^High^ and Sca-1^Low^ fractions by fluorescence activated cell sorting (FACS). The two fractions were treated with 50 or 100 uM Rosiglitazone (Rosi), and **A.**, cell proliferation and **B.**, expression of adipocyte-marker FABP4 by qRT-PCR, were measured. **C.** Relative fold change in mRNA expression of PPARγ measured by qRT-PCR relative to actin as a control. **D.** Flow cytometric analysis of membrane Sca-1 expression of phycoerythrin-labeled (Sca-1-PE) mOS-482 cells before and after treatment with 100 μM Rosi for 72 hours. The histogram shows mean fluorescence intensity of the indicated cells. Y axis is maximum mean fluorescence intensity. X axis is IgG-phycoerythrin stained cells (-phycoerythrin-conjugated - anti Sca-antibody).

We determined whether TZD treatment differentially affected the Sca-1/Sox2 Hi- and Sca-1/Sox2 Lo- expressing cells. Cells were FACS-sorted based on Sca-1 expression, treated with Rosi and evaluated for growth and expression of adipocytic genes. As seen in Figure 3A, the cells with high-expression of Sca-1 show concentration dependent growth inhibition with Rosi treatment that is not evident in Sca-1 negative cells. Sca-1 Hi cells exhibit concomitant induction of FABP4 mRNA, a marker of adipogenesis while there is no induction of FABP4 gene expression in the Sca-1 Low cells treated with Rosi (Figure 3B). We then examined if the two fractions have differing levels of PPARγ. Figure 3C shows that indeed the Sca-1 Hi cells express much higher levels of PPARγ accounting for their higher sensitivity to TZD. To assess whether TZD treatment affected the fraction of Sca-1 Hi cells, we determined the proportion of Sca-1 Hi cells before and after TZD treatment. Figure 3D shows that Rosi treatment led to a reduction in the Sca-1 Hi population from 75% to 56%. This suggests that the effects of TZD on growth, proliferation and adipogenesis seen within the entire tumor cell population are specifically attributable to the cancer stem cell fraction which expresses higher levels of PPARγ and that TZD treatment reduces the proportion of CSCs in the tumor population.

### Rosiglitazone decreases proliferation and induces adipogenesis in a subcutaneous xenograft model of osteosarcoma

**Figure 4:**
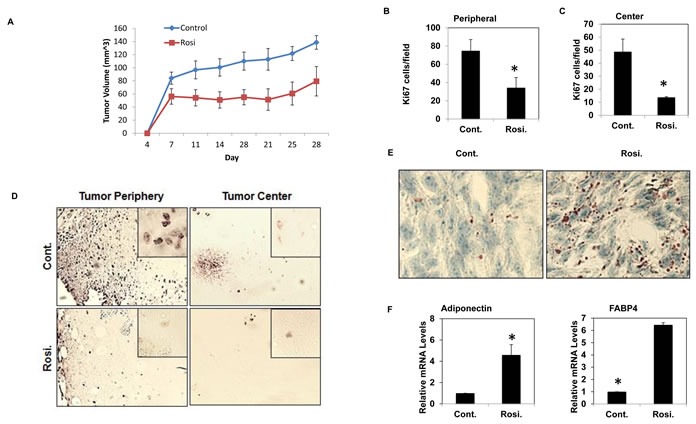
TZD treated mice have reduced tumor volume and increased adipogenesis NOD-SCID female mice (8 weeks old) were transplanted with 10^5^ mOS-482 cells and subsequently administered vehicle or Rosiglitazone (Rosi) orally five times a week for three weeks after implantation. **A.** Average tumor volume of treatment and control groups over time. **B.**, **C.**, **D.** Cell proliferation by Ki67 staining showing representative image of decreased Ki67 positive cells at the periphery and center of the tumors in Rosi treated tumors (mag 10X, inset- 40X). 10 fields for each area (*n* = 6) were counted. *, *P* < 0.05. **E.**, **F.** Oil Red O staining of the tumors shows increased adipogenesis in the treatment groups. Representative image at 40X is shown. mRNA expression of adipogenic genes in Rosi treated tumors compared to control by qRT-PCR (*n* = 4). *, *P* < 0.05.

To further determine the feasibility of DT, we investigated the effect of TZD treatment on osteosarcoma *in vivo* via a tumorigenesis assay in NOD/SCID mice. Mice were implanted subcutaneously with 100,000 mOS-482 cells and two treatment groups were assigned with seven mice per group. Mice were administered Rosiglitazone (100mg/kg) orally five times a week for four weeks upon cell implantation and tumor volume was measured bi weekly for 4 weeks. The dosage was selected in line with previously published studies [32, 33]. Figure 4A shows that Rosi treatment significantly delayed tumor growth as evidenced by reduced tumor volumes over the same time period. To determine proliferation, tumor sections were immunostained with an antibody to Ki67, a marker of proliferation. Rosi treatment led to a reduction of Ki67 positive cells on both the periphery and center of the tumors (Figure 4B-4D). The harvested tumors were also stained to monitor adipocytic differentiation. Figure 4E, 4F shows increased Oil Red O staining in the treatment group and consistent with this finding is the increased expression of adipocyte-marker genes, adiponectin and FABP4. Rosi treatment is known to increase bone marrow adipocytes. [34]. We also found that Rosi treated animals had increased marrow adipocytes (SI8). In line with the *in vitro* data, tumors from Rosi-treated animals did not show any increased apoptosis (SI8). Together, these experiments indicate that the effect of TZDs on osteosarcoma cells *in vitro* can be replicated *in vivo* where Rosi reduces cell proliferation and stimulates adipogenesis.

### Rosiglitazone decreases tumor bone volume and surrounding bone in an intrafemoral tumor model

**Figure 5:**
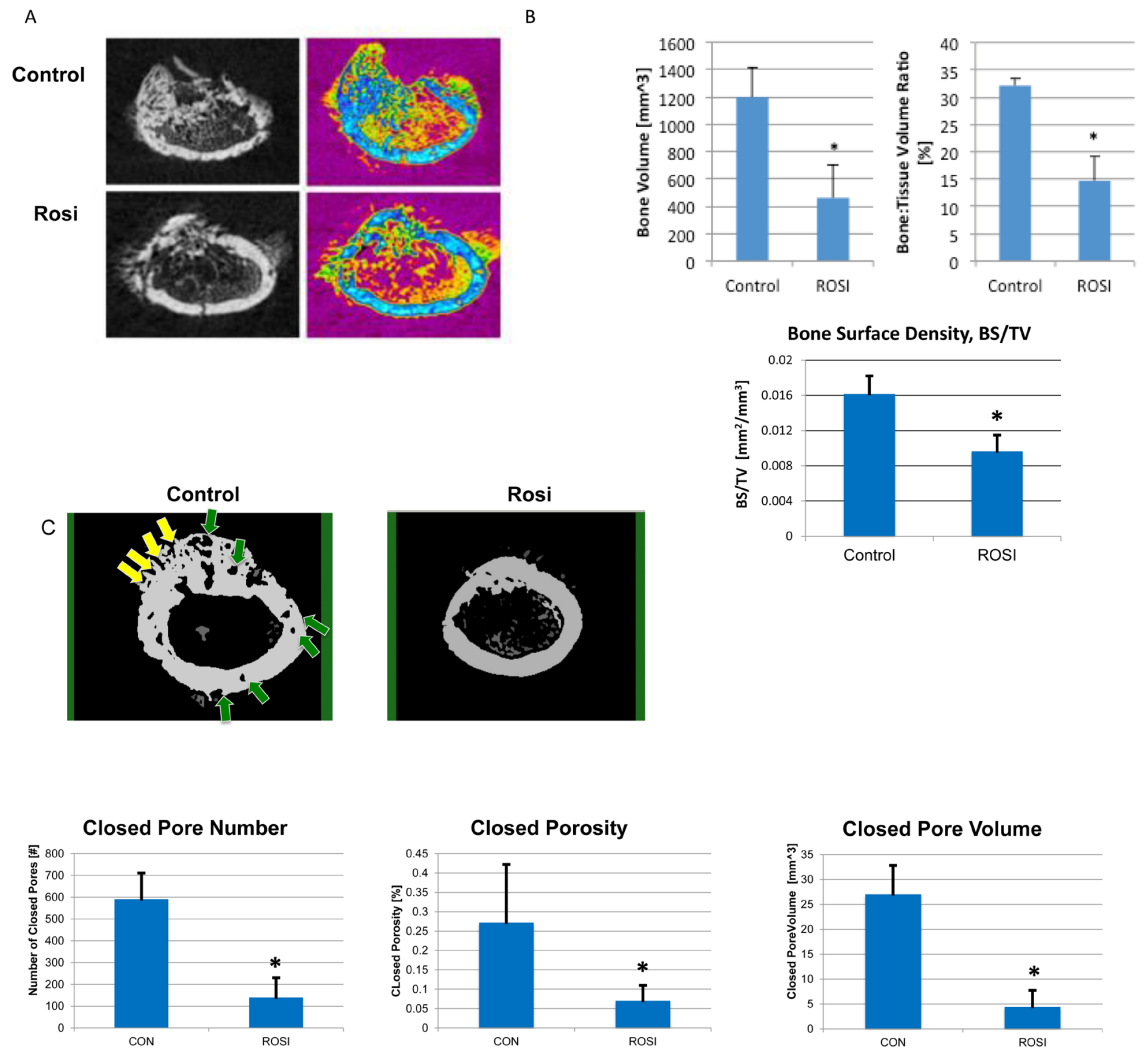
Rosiglitazone affects osteosarcoma tumor size and bone mineralization in orthotopic bone xenografts Four NOD/SCID mice per group were injected with 6×10^5^ mOS-482 cells into a unilateral distal femur. Mice were fed vehicle (control), or Rosiglitazone (Rosi) 100mg/kg from once daily, for 5 days per week from the start of implantation. Femurs were harvested at 3-weeks and cross-section images obtained by microCT. **A.** 2D reconstructed distal femoral cross-sectional images from control and Rosi treated animals in gray scale (left) and color-coded (right) to highlight decreased mineralized tumor tissue (blue) in the Rosi treated group. **B.** Total Bone Volume (BV), was also measured and found to be reduced by Rosi treatment, this value was then normalized to total volume (TV), from which the bone volume fraction was calculated (BV/TV), which showed a similar reduced level following Rosi treatment **C.** Porosity and bone surface density measurements. Representative MicroCT images of cortical bone from control and Rosi-treated femurs. Yellow arrows point to open pores and green arrows point to closed (intracortical) pores. Graphs show the porosity measurements, expressed as Closed Pore Number, Closed Porosity Closed Pore Volume. Average measurements from 4 animals per group *p < 0.05.

TZDs are known to have effects on bone and the MSC niche [34]. While the sub-cutaneous tumor model demonstrates that Rosi treatment reduces tumor growth, it does not capture the role of the specific tumor microenvironment in osteosarcomas. Bone tumors are unique in that they are in close contact with the MSC/osteoclast niche and cause extensive osteolysis of surrounding bone leading to additional complications for patients. We determined whether Rosi affected the bone content of the tumor, and the surrounding bone quality. mOS-482 cells were implanted orthotopically into the femurs of NOD/SCID mice and animals were administered vehicle or 100 mg/kg Rosi orally from the start of implantation as described in Figure 5A. After 3 weeks, the affected limbs were harvested and microCT imaging was used to analyze changes in mineralized morphology of the tumor [35]. Rosi treated mice showed a 62% decrease in tumor-specific bone volume fraction (BV/TV), and a 41% decrease in tumor-specific bone surface density (BS/TV) compared to control tumors (Figure 5A 5B). Extra-cortical bone tumor volume (blue areas) within the tumors is decreased overall in the Rosi-treated group.

We also assessed bone microarchitectural changes using a series of porosity measurements. Porosity is inversely proportional to several mechanical properties of bone such as strength and stiffness [36]. Porosities in the cortical bone (green and yellow arrows) were observed to be significantly reduced in the Rosi group compared to controls (Figure 5C). Quantitatively, the reduction in ‘number of pores’, ‘overall closed porosity’ and ‘pore volume’ all reflect the reduced tissue degradation that was present in the Rosi treated group (Figure 5D). Lipid accumulation in the bone marrow was also apparent in Rosi treated animals (not shown). These findings (discussed later) suggest that Rosi treatment can improve surrounding bone quality in an orthotopic model of osteosarcoma.

We also found that rosiglitazone treatment delayed initial dissemination of Saos2-LM7 human cells in an intravenous injection model of metastases (SI9).

### Rosiglitazone induces adipogenesis genes and reduces cell cycle-related and YAP target gene expression

**Figure 6:**
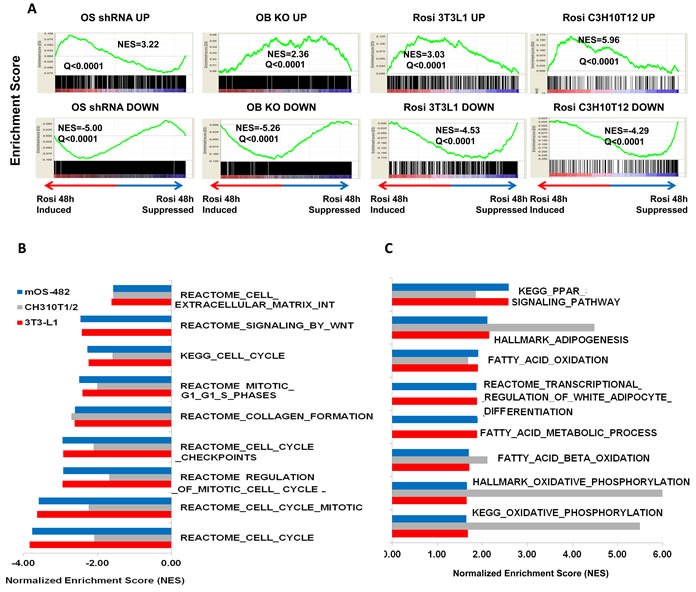
Gene expression analysis of rosiglitazone-treated cells **A.** Gene Set Enrichment Analysis (GSEA) plots. GSEA plots showing normalized enrichments scores (NES) for Rosi induced and suppressed genes in mOS482 osteosarcoma cells with genes up or down regulated by knockdown of Sox2 in osteosarcoma cells (OS shRNA UP and DOWN), osteoblasts (OB KO UP and DOWN), as well as Rosi-treated 3T3L1 preadipocytes (Rosi 3T3L1 UP and DOWN) and C3H10T1/2 cells (Rosi C3H10T1/2 Up and DOWN). **B.** Common down-regulated pathways in mOS482, C3H10T1/2 and 3T3L1 Rosi-treated cells. **C.** Common up-regulated pathways in mOS482, C3H10T1/2 and 3T3L1 Rosi-treated cells.

To determine the TZD-induced gene expression profile in osteosarcoma, mOS-482 cells were treated with Rosiglitazone (50uM for 24 and 48-hours) in triplicate and processed for RNA sequencing (RNA-SEQ) analysis as described in the methods. We have previously described a Sox2-regulated gene signature in osteoprogenitors and osteosarcoma cells where we found that Sox2 promotes cell cycle and stemness-related genes and suppresses Wnt signaling [17, 37]. These expression profiles were compared using Gene Set Enrichment Analysis (GSEA) with the Rosi induced gene expression profiles. GSEA analysis in Figure 6A shows a significant correlation of Rosi-induced genes with those repressed by Sox2 both in osteosarcoma (OS) and in normal osteoprogenitor (OB) cells. This finding fits with the notion that TZDs act on stem cells and repress stemness properties in osteosarcoma cells, as we have shown is achieved by the deletion of Sox2.

We also compared gene expression profiles of Rosi treated osteosarcoma cells with previously published gene expression data of Rosi treated cells (C3H 10T1/2 MSCs and 3T3L1 preadipocytes at 48 hours). [38]. As indicated in Figure 6A, the Rosi transcriptome response in our experiment matches significantly and in the same direction (Q < 0.25) with the Rosi response in C3H 10T1/2 MSCs and 3T3-L1. Analysis of common pathways analysis shown in Figure 6B point to a down regulation of pro osteogenic pathways such as Wnt signaling and collagen synthesis with a concurrent upregulation of lipid metabolism and fatty acid synthesis pathways. Common signatures of induced genes include genes required for adipogenesis and fatty acid metabolism (e.g. PDK4 and PPARγ) (Figure 6C). Furthermore, expression of osteogenic differentiation markers such as osteoglycin was strongly downregulated after Rosi treatment in all three cell lines. These findings suggest that osteosarcoma cells retain the ability to respond to PPARγ activation as do their normal counterparts and corroborates the reciprocity of the osteo-adipo lineages [39, 40]. Importantly, TZD treatment of OS cells leads to a downregulation of tumorigenic/cancer-related genes in osteosarcoma cells. Thus gene expression analysis also indicates that TZD treatment drives adipogenesis in osteosarcoma cells and reduces their growth and tumorigenic properties.

### Regulation of YAP1 by TZDs

**Figure 7:**
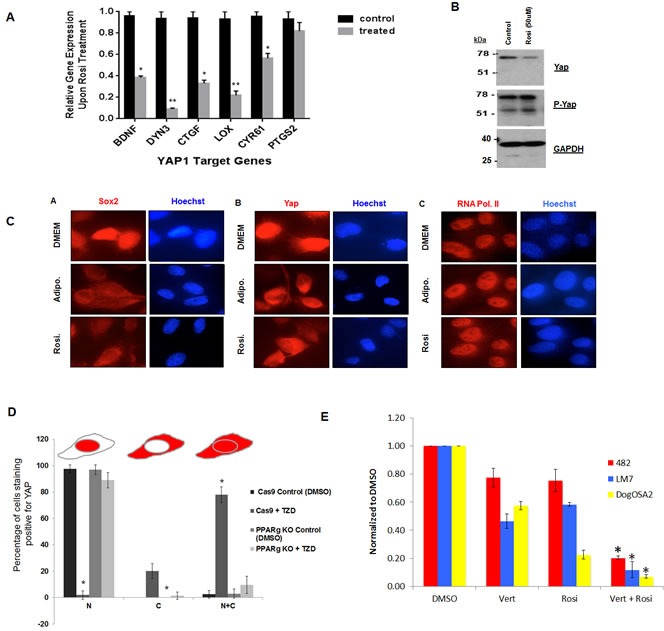
Rosiglitazone decreases YAP nuclear localization and YAP-dependent transcription in osteosarcoma cells **A.** Expression of canonical YAP target genes in mOS-482 cells treated with 100 uM rosiglitazone for 48 hours. **B.** Western blot of YAP and phospho-YAP **C.** Immunofluorescence using Sox2, YAP and RNA Pol II antibody on mOS-482 cells treated with Rosi or adipogenic media for 48 hours. Images were taken using a Leica DM5500 immunofluorescence microscope at 63x magnification. **D.** YAP localization by TZD is dependent on PPARγ. Quantification of YAP immunostaining in CAS9 (control) and PPARγ knockout cells treatedwith 50 μM Rosi for 24 hours. Images were taken using a Leica DM5500 immunofluorescence microscope at 63x magnification. Ten fields in each condition were counted and average percentage of cells showing exclusively nuclear (N), exclusively cytoplasmic (C), or both nuclear and cytoplasmic (N+C) is shown in histogram. *p < 0.05 **E.** Growth of mOS482 (mouse), LM7 (human) and OSA2 (dog) osteosarcoma in medium supplemented with (DMSO), verteporfin - vert (250 nM), Rosi (10 μM -for mouse and 50 μM for human and dog) or both Rosi and Vert cells for 48 hours. Graph shows growth relative to DMSO control averaged from three replicates in each condition.

We also observed in the gene expression analysis that genes that are bonafide targets of YAP such as BDNF, DYN3, CTGF LOX and CYR61 have reduced expression upon TZD treatment only in osteosarcoma cells. We had previously validated a set of YAP targets by knockdown of YAP in osteosarcoma [17], and we verified that these genes are indeed down regulated by TZD treatment by qRT-PCR (Figure 7A).

YAP is the downstream effector of the tumor suppressive Hippo signaling pathway that is necessary for the regulation of organ size and cell proliferation [16, 41]. YAP acts as a transcriptional co-activator by associating with TEA-domain family member (TEAD) and subsequently regulating expression of target genes necessary for cell proliferation. YAP is transcriptionally inactivated by Hippo signaling via phosphorylation and sequestered in the cytoplasm [42, 43]. We have previously shown that Hippo signaling is repressed by SOX2 in osteosarcomas and these tumors have high YAP that is required for tumorigenesis [17]. We therefore determined whether SOX2 and YAP are affected by TZD treatment. While we did not detect any differences in Sox2 or YAP RNA levels (data not shown), the protein level of YAP was reduced in TZD-treated cells while YAP phosphorylation was enhanced (Figure 7B).

Immunofluorescence of SOX2 and YAP in mouse osteosarcoma cells shows that these transcription factors lose their nuclear localization and are detectable in the cytoplasm under adipogenic conditions and that treatment with Rosi caused a similar change in localization (Figure 7C), in line with the increased phosphorylation of YAP. RNA Pol II expression remained confined to the nucleus suggesting that Rosi treatment does not compromise the integrity of the nuclear membrane in osteosarcoma cells. Similar results were seen with human LM7 and OS182 cells and with pioglitazone treatment (data not shown). Together, these data highlight an additional mechanism in TZD-induced adipogenesis where the transcriptional activity of YAP is decreased upon cytoplasmic sequestration. The effects of TZDs on YAP localization are abrogated in PPARγ knockdown OS cells in which YAP remains exclusively nuclear upon Rosi treatment, suggesting that PPARγ expression is required for the effect on YAP localization and transcriptional activity (Figure 7D). This finding is corroborated by a decrease in expression of canonical YAP target genes and suggests that TZDs affect YAP-dependent transcription and activation of Hippo signaling in osteosarcoma cells. Importantly Rosi shows synergistic growth inhibitory effects when combined with verteporfin, an inhibitor of YAP-TEAD mediated transcription [44] in mouse human and dog osteosarcoma cells (Figure 7E). These data demonstrate the importance of reducing YAP function in the antitumor effects of TZDs in osteosarcoma.

### A Wnt-Hippo-PPARγ gene expression signature predicts outcomes in osteosarcoma

From the results presented it is apparent that a TZD mediated differentiation therapy would target specifically the OS stem cell population. Our previous analysis had revealed that Wnt signaling is low in CSCs that express Sox2 and form osteospheres. Sox2-depleted cells have higher Wnt signaling, as evidenced by an increase in canonical Wnt targets, such as TIMP3 [15, 37]. We also found that YAP target genes such as CTGF and CYR61 are highly expressed in the CSCs and are reduced in Sox2 and YAP depleted cells [17]. Thus high Wnt signaling and low YAP target genes (high Hippo signaling) mark a population of differentiated, less stem-like osteosarcoma cells.

**Figure 8:**
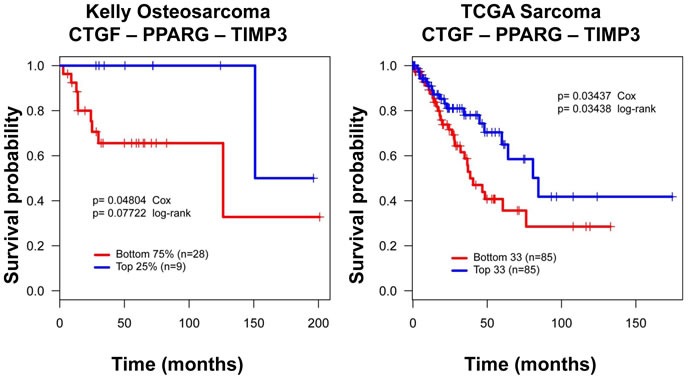
A CTGF-PPARγ-TIMP3 signature correlates with clinical outcome in osteosarcoma Kaplan-Meier survival curves for CTGF, PPARγ and TIMP3 illustrating that higher CTGF, and PPARγ and low TIMP3 expression correlates with worse outcomes, and the reverse pattern of expression correlates with better outcome in two independent datasets. Distribution of patients for this data set has been previously published. Survival probability and P values calculated using Kaplan-Meier and Cox proportional hazards methods. DFS = Disease-free survival in Kelly, OS = Overall survival in TCGA.

Based on these *in vitro* results, we developed a dichotomized gene expression signature -(CTGF^Low^ PPARγ^Low^ and TIMP3^High)^ or (CTGF^High^ PPARγ^High^ and TIMP3^Low^) and correlated the signature to good and poor outcomes (time in days from diagnosis) by conducting Kaplan-Meier analysis. Overall survival data from The Cancer Genome Atlas (TCGA), and disease-free survival data from Kelly et al. were used for this analysis [45]. As shown in Figure 8, patients with CTGF^Low^ PPARγ^Low^ TIMP3^High^ tumors had higher overall survival, or disease free survival in the two databases respectively whereas patients with CTGF^High^ PPARγ^High^ TIMP3^Low^ cancer had poorer prognosis. This is probably because high TIMP3 and low CTGF and PPARγ expression indicates a more differentiated and less stem-like osteosarcoma sub-type with high Wnt and Hippo signaling. In this view, patients with the CTGF^High^ PPARγ^High^ TIMP3^Low^ signature may be better candidates for TZD-induced differentiation therapy.

## DISCUSSION

Osteosarcomas are genetically highly heterogeneous with multiple oncogenic drivers which has been a major obstacle in developing targeted therapy for this tumor type [46–49]. In this study, we present proof of principle for differentiation therapy for osteosarcoma which relies on the use of TZDs to stimulate adipogenesis in these cells. Across species, osteosarcoma cells demonstrate growth inhibition and enhanced adipogenesis when treated with the TZDs, Pio or Rosi. Additionally we report a novel mode of action of TZDs in osteosarcoma in promoting the nuclear exclusion of SOX2 and YAP transcription factors. TZDs also decreased tumorigenicity of osteosarcoma cells in xenotransplantation assays. The effects of TZDs are independent of the origins or driver mutations, suggesting the broad therapeutic potential of these agents for the treatment of osteosarcoma. Gene expression analysis of TZD -treated cells led to the identification of a signature that is predictive of patient outcomes. This study provides pre-clinical rationale for an osteosarcoma differentiation therapy with a targeted agent.

### PPARγ activation in cancer stem cells has anti-neoplastic effects in osteosarcomas

DT represents an attractive alternative to conventional treatment for osteosarcomas by inducing terminal differentiation of CSCs. By utilizing TZDs that function as high-affinity agonists of PPARγ [20], we demonstrate that TZD-induced activation of this nuclear receptor exerts anti-proliferative effects via promoting adipogenic differentiation in osteosarcoma cells. Unlike troglitazone that requires RXR activation [50], we find that the effect of TZD in osteosarcomas cells does not require exogenous RXR ligands. PPARγ-null osteosarcoma cells do not respond to TZDs, thereby highlighting that PPARγ is essential for anti neoplastic effects of TZDs.

The Sca-1- expressing CSC population of osteosarcoma that are responsible for tumor seeding, recurrence and metastasis is more responsive to TZD treatment These cells have elevated expression of PPARγ which probably accounts for this effect. In clinical practice, however, TZD treatment would likely be used as an adjuvant or neo-adjuvant treatment modality, along with surgery and/or chemotherapy. This two-pronged strategy would ensure tumor debulking (through surgery or chemotherapy) and CSC depletion, such that relapse through persistence of osteosarcoma stem cells is minimized.

We also found that cotreatment with the liver-derived hormone FGF21 that stabilizes PPARγ, enhances TZD-induced adipogenesis. [30]. FGF21 also acts on bone marrow mesenchymal cells to promote differentiation into adipocytes rather than osteoblasts.[28] Future experiments will assess this hormone's effect on osteo-adipo lineage fate and if its effects are similarly potentiated by concomitant treatment with TZDs.

### TZDs mimic reactivation of Hippo signaling in osteosarcoma cells

Hippo signaling has been identified as a tumor suppressive pathway in tumors of both epithelial and mesenchymal origin cancers. When active, the pathway phosphorylates and suppresses the transcriptional co-activators YAP and TAZ/WWTR1. We have previously demonstrated that osteosarcomas have high YAP activity, which maintains CSCs. Loss of YAP decreases the stem cell fraction and restores osteogenic differentiation [17]. The other transcriptional co-activator, TAZ/WWTR1, targeted by the Hippo signaling pathway is expressed at very low levels in mOS-482 cells.

Treatment of osteosarcoma cells with TZDs leads to YAP phosphorylation and nuclear exclusion, with a decrease in canonical YAP target genes, suggesting that TZDs exert their tumor suppressive activity in part through a mechanism akin to activation of the Hippo pathway. While the exact mechanism of this re-activation remains to be deciphered, experiments in 3T3-L1 cells suggest that TZD treatment activates Lats2, one of the upstream kinases that phosphorylate YAP [51]. Hippo signaling is probably not the only mechanism of action of TZDs. TZDs synergizes with verteporfin, an agent interfere with YAP-TEAD interaction, to block osteosarcoma growth. This points to a novel combination of drugs to target osteosarcomas. Like the TZDs, verterporfin is also in clinical use thus providing a rationale for drug repurposing.

### TZDs induce a gene signature in osteosarcoma cells that is predictive of patient outcomes

RNA-Seq analysis of TZD treatment of mOS cells re-confirmed the reciprocal antagonistic relationship between Wnt and PPARγ signaling in the mesenchymal lineage. Even more striking was the down regulation of Hippo targets in the osteosarcoma cells. This could be due to the fact that osteosarcoma cells are addicted to YAP expression that needs to be down-regulated for adipogenic differentiation to occur, unlike normal mesenchymal and pre-adipocytic cells.

The gene expression signature we developed reflects the unique interplay between Hippo, Wnt and PPARγ signaling. Low TIMP3 (Wnt target), and high CTGF (YAP target) and PPARγ expression suggest a more aggressive osteosarcoma sub-type with low Wnt and Hippo signaling, and a higher propensity to adipocytic differentiation as reflected by high PPARγ expression. Interestingly, in both the databases queried, we found that TIMP3^High^ CTGF^Low^ PPARγ^Low (^more differentiated and less aggressive osteosarcoma sub-type) cancers was associated with better survival using two independent cohorts. This analysis recapitulates the antagonistic relationship described between Wnt and PPARγ signaling in normal bone, in the context of osteosarcoma development.

In view of the results presented above, it may seem counterintuitive that high levels of an adipogenic factor (PPARγ), low activity of an antiadipogenic, pro-osteogenic pathway (Wnt), and low Hippo function would characterize highly aggressive tumors with poor prognosis. Low Wnt activity, high levels of PPARγ and low Hippo pathway activity characterize the CSC fraction of the osteosarcoma cell population, where high Sox2 expression antagonizes the Hippo pathway, represses the Wnt pathway and regulates PPARγ expression. We therefore believe that the survival analyses reflect the fact that the least differentiated, most aggressive tumors contain a larger proportion of CSC than the more benign tumors, a conclusion previously reached by an analysis of lung and breast cancers [52]. In this view the most aggressive osteosarcomas would be the ones that could mostly benefit from a CSC targeted differentiation therapy. Of particular interest is the fact that this signature is predictive of survival in two different data sets and is independent of the tumor heterogeneity of osteosarcomas.

Despite the therapeutic potential of TZDs for DT, their clinical use has been challenged because of their safety profile with long-term use as in diabetes.[19] This includes significant weight gain, bone loss, and increased risk of fracture and bladder cancer. However, a more recent and larger study has determined that pioglitazone use was not associated with an increased risk to bladder cancer [53]. The relationship between exposure duration and adverse effects makes it important to modulate treatment to maximize the benefit-risk ratio. Unlike the use of TZDs for the treatment of T2D that is typically low-dose and long-term, we anticipate that TZD use in DT would be high-dose, short-term in turning aggressive tumor cells to less proliferative fat cells. This along with the emergence of second-generation TZDs maintains its promising outlook [19]. Thus DT via TZD treatment may be a potential adjuvant therapy for osteosarcoma and other cancers of the mesenchymal lineage. DT would also be applicable to other cancers such as gliomas where CSCs have been identified.

## MATERIALS AND METHODS

### Cell culture

The mouse osteosarcoma cell line mOS-482 was attained from a spontaneous osteosarcoma and previously described [7]. The human osteosarcoma cell line OS-187 and Saos-2-LM7 were obtained from Dr. N. Gordon, and Dr. E. Kleinerman respectively, MD. Anderson Cancer Center, Houston, TX. Cells were maintained at 37°C in Dulbecco's Modified Eagle Medium (DMEM) supplemented with 10% fetal bovine serum (FBS) and antibiotics.

### Survival analysis

Survival analysis was carried out for a proposed subset of genes using the Cox-proportional hazards models as implemented *survival* package in R. We employed the following patient cohorts: the sarcoma patients cohort collected by The Cancer Genome Atlas (TCGA; https://tcga-data.nci.nih.gov/tcga/) and an osteosarcoma cohort collected by Kelly at al. For a gene signatures and a patient cohort, using a previously proposed methodology [54]: by computing an activity score for each patient in the cohort as follows: all genes are z-score transformed, then for each sample we add the z-score for up-regulated genes and subtract it for down-regulated genes. Specimens were sorted by activity score, then survival association was evaluated using R.

### *In vitro* scratch assay

10^6^ cells were plated in six-well plates and grown to confluency. Cells were subsequently serum-starved and a scratch was made on the monolayer using a pipette tip. Cells were then treated with TZD prepared in serum-free DMEM for 18h cells were briefly stained with Hoechst 33342. Images were taken using a Carl Zeiss AxioCam MRc camera.

### *In vivo* tumorigenicity assay

Tumorigenesis studies were performed at the Antitumor Assessment Facility at Memorial Sloan Kettering Cancer Center (IACUC Protocol Number A3311-01). 10^6^ cells were injected subcutaneously into female NOD/SCID mice. Animals were monitored and weighed twice weekly. Tumor volumes were measured by Vernier calipers. Two treatment groups were established with ten mice per group. Mice were gavage-fed vehicle or rosiglitazone (100 mg/kg) five days a week for of four weeks. For orthotopic intrafemoral tumor assays, 10^6^ cells mOS-482 cells were injected intrafemorally above the knee joint in 6-8 week old NOD-SCID female mice. 5 mice in each group were fed orally with vehicle or rosiglitazone (100mg/kg) at the start of transplantation (five days a week for three weeks) and were monitored for tumor growth by palpation and weekly by X-ray. Upon sacrifice femurs were analyzed by micro CT (Skyscan) at the NYU School of Dentistry micro CT core.

### *In vitro* growth assay

OS cells were plated at a density of 5,000 cells/well in 24-well plates in supplemented DMEM. Cells were treated with various concentrations of TZDs (Cayman Chemicals, stock = 100 mM) in triplicate prepared in DMSO solvent. FGF21 (stock = 2.4 mg/mL) dilutions were prepared in a 50% glycerol buffer. Cell counts were attained using a hemocytometer after 48 and 72 hours at 37°C.

### Immunohistochemistry

Tissues were fixed and embedded in paraffin. Paraffin-embedded sections were deparaffinized in Citrosolv and then rehydrated in an ethanol series. Antigen retrieval was performed at pH 6, 10mM sodium citrate buffer and slides were blocked in goat serum. Antibodies used - Ki67 antibody at 1:200 dilution (Thermo Scientific), SOX2 at 1:200 dilution (Millipore), or anti YAP at 1:400 dilution (Santa Cruz). Staining was visualized using a Vectastain Elite ABC Kit (Vector Labs). Control IgG was used as a negative control.

### Adipogenic differentiation

24- well plates were seeded at 50,000 cells/well in supplemented DMEM. Cells in duplicate wells were treated with adipogenic induction media and grown in a 37°C incubator for various time points over several days. Adipogenic medium contains 100μM indomethacin, 10μg/μl insulin, 100nM dexamethasone, and 250μM isobutylmethylxanthine. Adipogenesis was detected by staining with Oil-Red-O (Sigma).

### Western blotting

Cells were grown in supplemented DMEM in a 37°C incubator until confluent. The medium was changed every three days and lysed in radio-immunoprecipitation assay (RIPA) buffer containing protease inhibitors. Samples were kept on ice for thirty minutes and after centrifugation at 13,000 rpm for twenty minutes, the supernatants were collected. After determining the protein concentrations of the cell extracts using a Bio-Rad DC protein assay, samples were run on a SDS-PAGE gel. The separated proteins were transferred overnight to a polyvinylidene fluoride (PVDF) membrane at 4°C. The following day, the membrane was blocked via application of 5% nonfat dry milk in Tris-buffered saline with 0.05% Tween-20 (TBST) for an hour at room temperature. Membranes were probed with primary antibodies (all at 1:1000 dilutions) for SOX2 (Cell Signaling), β-catenin (Millipore) and PPARγ (Cell Signaling). Anti-tubulin antibodies were used as a normalization control. Following secondary probing with monoclonal anti-mouse or polyclonal anti-rabbit antibody probes, protein blots were visualized with an enhanced chemiluminesence detection reagent (Amersham), exposed to an X-ray film, and developed.

### Quantitative real-time RT-PCR

Total RNA was extracted using an RNeasy mini kit (Qiagen) and treated with DNase using the manufacturer's protocol. 0.5μg of purified RNA was reverse transcribed at 42 degrees for 65min using SuperScript II RT and Oligo(dT) as a primer in a final volume of 20μL. 2μL was used as a template for amplification using gene specific primers sets. RT-PCR was carried out on a Light Cycler Instrument using the DNA Master SYBR Green I dye intercalation assay (Roche). Actin was used as a normalization control.

### Transcriptomic data analysis

Total RNA from triplicate plates of control (DMSO) treated or Rosi treated cells was prepared using RNeasy columns. Illumina libraries and were prepared with the TruSeq protocol and sequenced on Illumina Hi Seq 2000 at the NYULMC Genome Technology Center. Reads were mapped with TopHat to the University of California Santa Cruz (UCSC) mouse genome mm10 genome assembly [55]. Gene expression was quantified with Cufflinks 2.0 software [55]; significant changes were assessed using T-test for statistical significance (p < 0.05) and fold change of 1.5, using the R statistical analysis system. Unsupervised clustering visualization was generated using R. Gene Set Enrichment Analysis (GSEA) was carried out using the GSEA software package [56]. We integrated the transcriptome profiling of C3H 10T1/2 MSCs and 3T3L1 response to Rosi generated by Rong et al; we derived the gene signatures of Rosi response using t-test (p < 0.05) and fold change exceeding 1.5x.

### Survival analysis

Survival analysis was carried out for a proposed subset of genes using the Cox-proportional hazards models as implemented *survival* package in R. We employed the following patient cohorts: the sarcoma patients cohort collected by The Cancer Genome Atlas (TCGA; https://tcga-data.nci.nih.gov/tcga/) and an osteosarcoma cohort collected by Kelly at al. For a gene signatures and a patient cohort, using a previously proposed methodology [54]: by computing an activity score for each patient in the cohort as follows: all genes are z-score transformed, then for each sample we add the z-score for up-regulated genes and subtract it for down-regulated genes. Specimens were sorted by activity score, then survival association was evaluated using R.

### Micro-CT analysis

Changes in localized bone microstructure were assessed using a microCT system (SkyScan 1172; Bruker microCT, Belgium) where projections (4000×4000 pixels) of the distal femur were acquired at a nominal isotropic resolution of 9 um. To reduce the variability in the beam intensity profiles across the image, the approximate centerline of each bone was aligned to the axis of rotation of the system. A 10W power energy setting (100 kV and 100mA) and a 0.5-mm aluminum filter were used to minimize beam hardening effects by filtering out low-energy photons. An alignment procedure and flat-field detector calibration were performed to minimize ring artifacts and increase signal-to-noise ratio. Then, 180° degree scans were performed with five X-ray projections acquired every 0.3 degrees, each with an exposure time of 1070 ms. These scanning parameters, were chosen in accordance with the guidelines for mCT analysis of rodent bone structure [35]. A modified back-projection reconstruction algorithm (v.1.6.5, NRecon, SkyScan; Bruker microCT, Belgium) [36] was used to generate cross-sectional images from the X-ray projections. Images were optimized and corrected for ring artifacts and further beam hardening correction was achieved using the NRecon software to check that the X-ray intensity profiles across the bone cross-section remained linear. Using manufacturer software (CtAn, Bruker microCT, Belgium) the tumor regions were re-oriented such that regions of interest (ROIs) could be defined and compared in the transverse plane. 3D parameters were assessed to describe the mineralized microstructure in the region: bone volume (BV), total volume (TV), from which the bone volume fraction was calculated (BV/TV), bone surface area (BS), as well as total porosity and more refined derivatives including closed porosity, open porosity and pore number/volume. The standard trabecular bone microstructural parameters are not reported here since this is a pathological bone formation system, and those measurements are not easily interpretable in this setting.

## SUPPLEMENTARY MATERIALS FIGURES


