# Supplementary material for: PPARγ agonists promote differentiation of cancer stem cells by restraining YAP transcriptional activity
Source: Oncotarget. 2016 Aug 12;7(38):60954–70. doi: 10.18632/oncotarget.11273 (PMC5308629; doi:10.18632/oncotarget.11273)
Supplement: Supplementary file 1 [file oncotarget-07-60954-s001.pdf]

PPARγ agonists promote differentiation of cancer stem cells by restraining YAP transcriptional activity

Supplementary Material

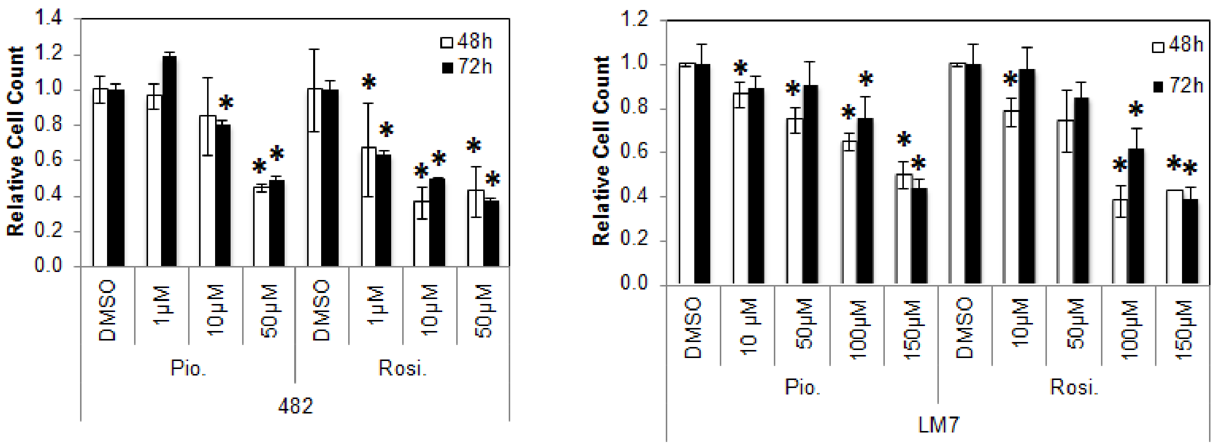

Supplementary Figure SI1. Pioglitazone also induces growth arrest in osteosarcoma.

mOS 482 (mouse) and LM7 (human) cells exhibit concentration-dependent growth arrest when treated with Pioglitazone (Pio) or Rosiglitazine (Rosi) for 48 and 72 hours (n=3; * $P<0.05$).

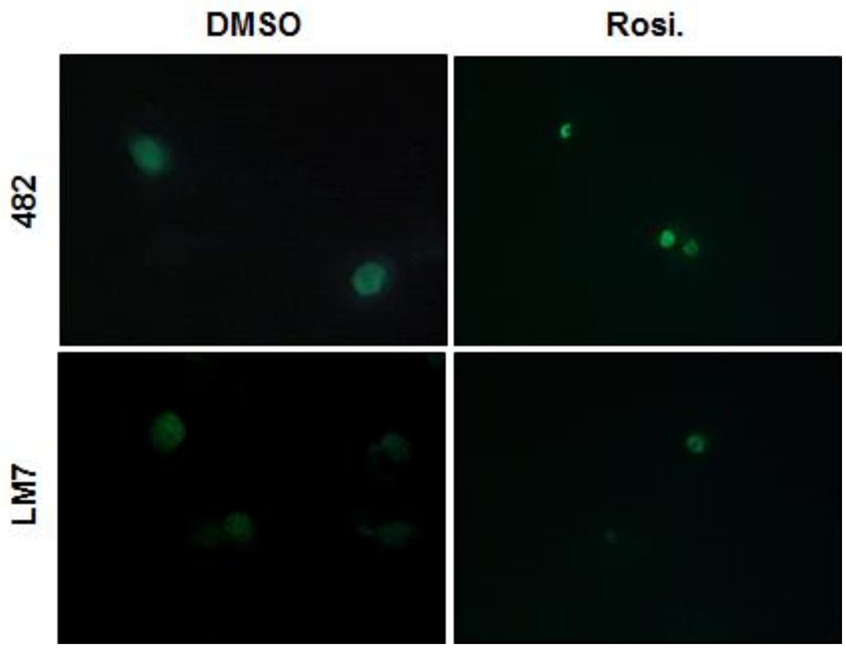

Supplementary Figure SI2. Rosi does not induce apoptosis in osteosarcoma.

TUNEL assays on 482 and LM7 cells treated for 48h with 50μM and 100μM of Rosi, respectively, showed no increase in the number of apoptotic cells. Images shown are at 40x magnification using a Carl Zeiss AxioCam MRc camera.

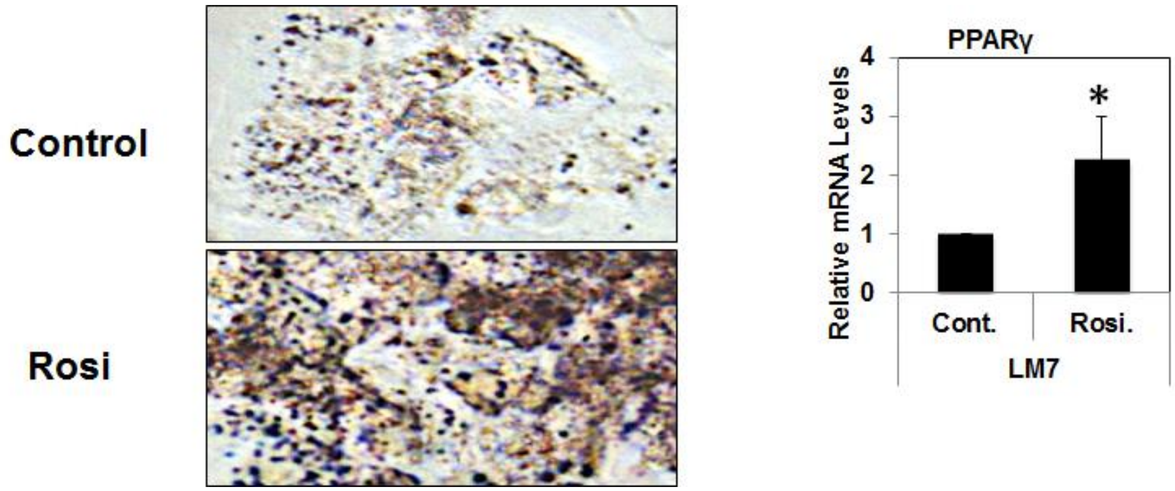

Supplementary Figure SI3. Rosi induces adipogenesis in human Saos2-LM7 cells.

Oil Red-O lipid stain of LM7 cells grown in adipogenic media (top panel) and the addition of Rosiglitazone 10uM (bottom panel) stained at 10-days. **(B)** Relative fold change in mRNA expression of PPARγ measured by qRT-PCR. * = $p < 0.05$.

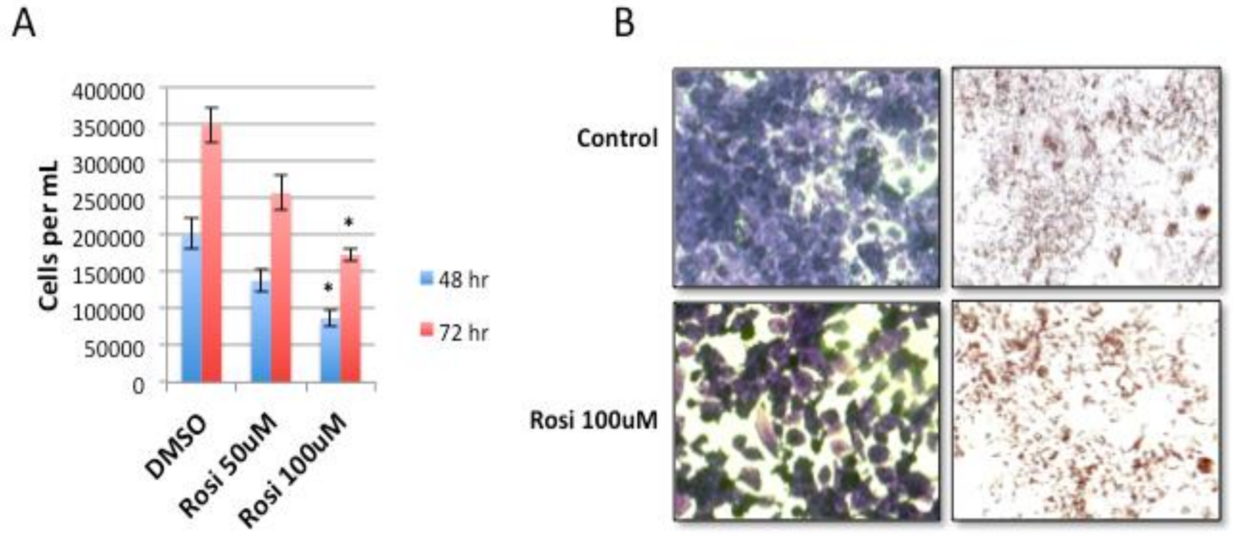

Supplementary Figure SI4: Rosiglitazone decreases growth and induces adipogenesis in a primary canine osteosarcoma cell line.

(A) Growth of dog OSA2 cells treated with DMSO, Rosiglitazone 50uM and 100uM for 48- and 72-hours. * = $p < 0.05$. (B) Differentiation assays with Dog OSA2 cells grown in regular media and treated with Rosiglitazone 100uM for 10 days. Stained with crystal violet (left panel) and Oil-Red-O (right panel). Mag 40X

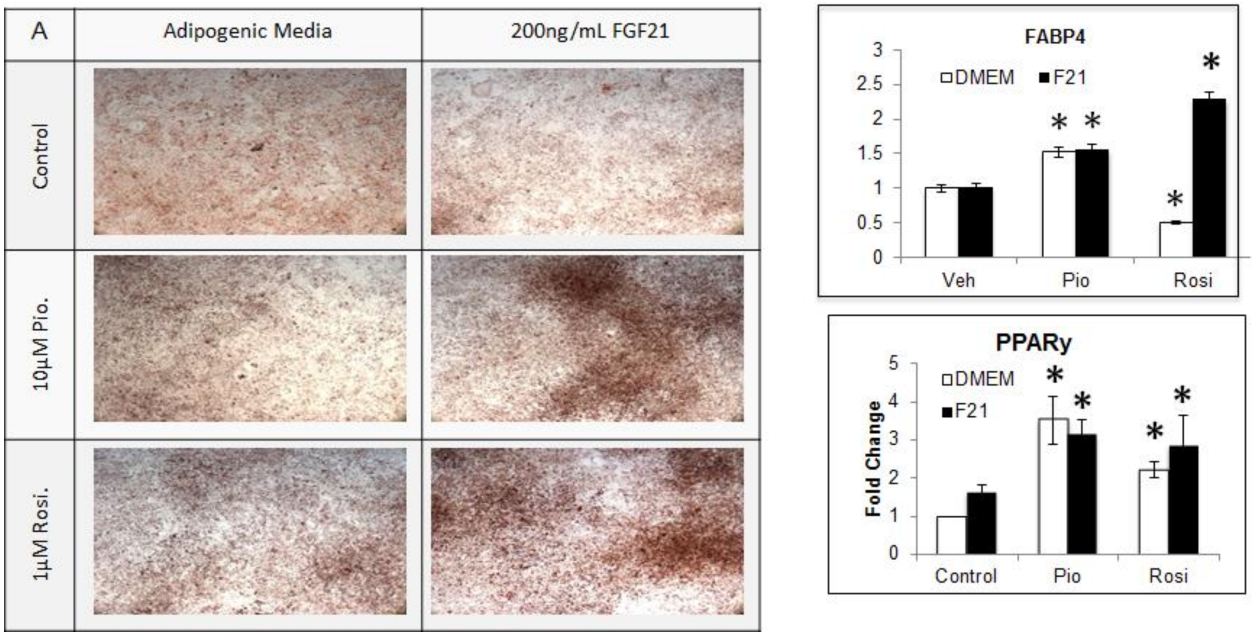

Supplementary Figure SI5: FGF21 synergizes with TZDs to induce adipogenesis in mOS-482 cells

mOS-482 cells treated in adipogenic media for 72 hours (control) in the presence of Pio (10µM), Rosi (1µM) singly or together with FGF21 (200 ng/mL). Enhanced adipogenesis is seen with the combination of TZD and FGF21 as evidenced by increased Oil Red O staining and qRT-PCR analysis of adipocyte-specific genes. QRT-PCR analysis (n=3). * = $p < 0.05$.

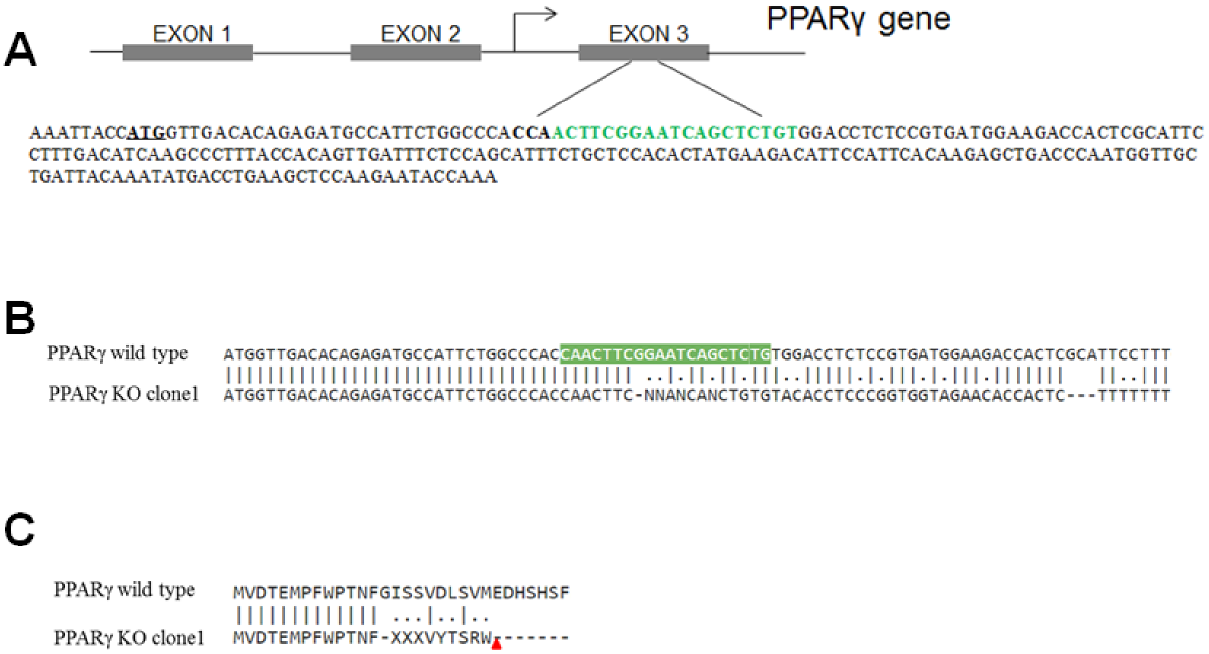

Supplementary Figure SI6

Confirmation of PPARγ deletion in mOS cells by Sanger sequencing . A. Green highlighted sequence in Exon 3 of PPARγ is the gRNA. B Deletion in PPARγ knockout (KO) clone 1 confirmed by Sanger sequencing. C Putative truncated protein sequence in PPARγ KO OS cells.

Control

Histogram Statistics

File: v mos482 PE Unstain02122016.001
Sample ID:
Panel: Untitled Acquisition Tube List
Gated Events: 9842
X Parameter: FL2-H (Log)

Log Data Units: Linear Values
Tube: Untitled
Gate: G1
Total Events: 10876

Marker	Left, Right	Events	% Gated	% Total	Mean	Geo Mean	CV	Median	Peak Ch
All	1, 9910	9802	100.00	90.13	2.33	1.89	108.50	1.73	1
M1	9, 9910	127	1.30	1.17	17.58	15.18	62.68	12.63	9
M2	85, 5935	0	0.00	0.00	***	***	***	***	***

DMSO

Histogram Statistics

File: v mos482 PEcnt untr02122016.002
Sample ID:
Panel: Untitled Acquisition Tube List
Gated Events: 9709
X Parameter: Sca1-PE (Log)

Log Data Units: Linear Values
Tube: Untitled
Gate: G1
Total Events: 11029

Marker	Left, Right	Events	% Gated	% Total	Mean	Geo Mean	CV	Median	Peak Ch
All	1, 9910	9611	100.00	87.14	771.52	420.65	105.27	504.81	537
M1	9, 9910	9596	99.84	87.01	772.71	423.32	105.12	509.37	537
M2	85, 5935	8356	86.94	75.76	871.65	593.83	90.50	604.30	537

Rosi

Histogram Statistics

File: v mos482 PE100uM Ro02122016.004
Sample ID:
Panel: Untitled Acquisition Tube List
Gated Events: 10273
X Parameter: Sca1-PE (Log)

Log Data Units: Linear Values
Tube: Untitled
Gate: G1
Total Events: 12112

Marker	Left, Right	Events	% Gated	% Total	Mean	Geo Mean	CV	Median	Peak Ch
All	1, 9910	10166	100.00	83.93	436.87	174.31	134.95	209.08	495
M1	9, 9910	9942	97.80	82.08	446.57	187.92	132.70	220.67	495
M2	85, 5935	6786	66.75	56.03	634.43	424.17	98.69	421.70	495

Supplementary Figure SI7:

Detailed statistics of flow cytometry analysis to detect Sca-1-PE positive cells. Panels include untreated, DMSO- and Rosi-treated cells. % Total shows decrease with Rosi treatment.

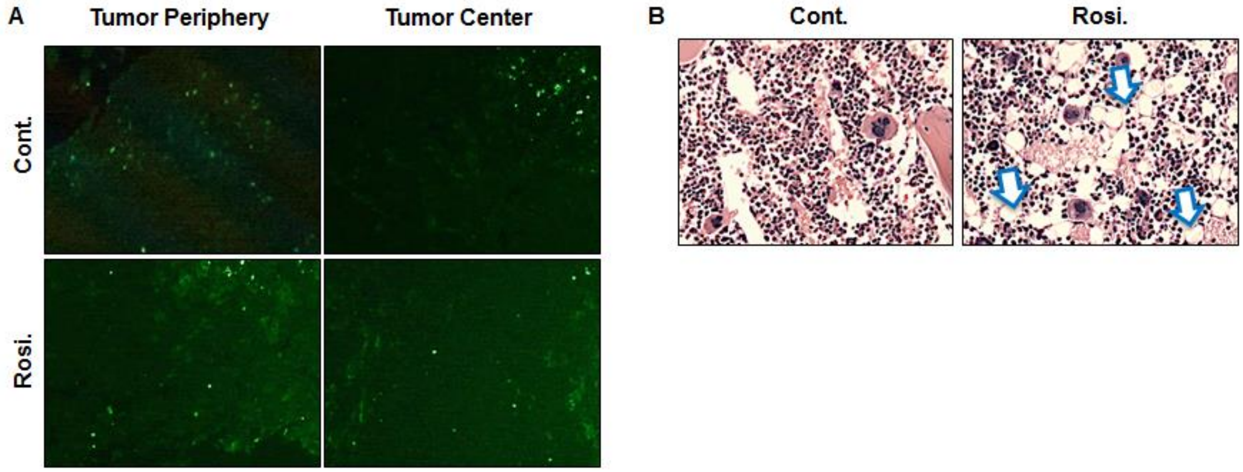

Supplementary Figure SI8. Rosi treatment in vivo does not increase osteosarcoma apoptosis and increases bone marrow fat production.

(A) TUNEL assay show no change in cell death in the xenograft tumors, both at the periphery and center, after Rosi-treatment. Pictures were taken using a Leica DM5500 microscope and are at 10x magnification. (B) Bone marrow in Rosi-treated mice femurs exhibit increased adipocytes indicated by blue arrows. Images -20x magnification.

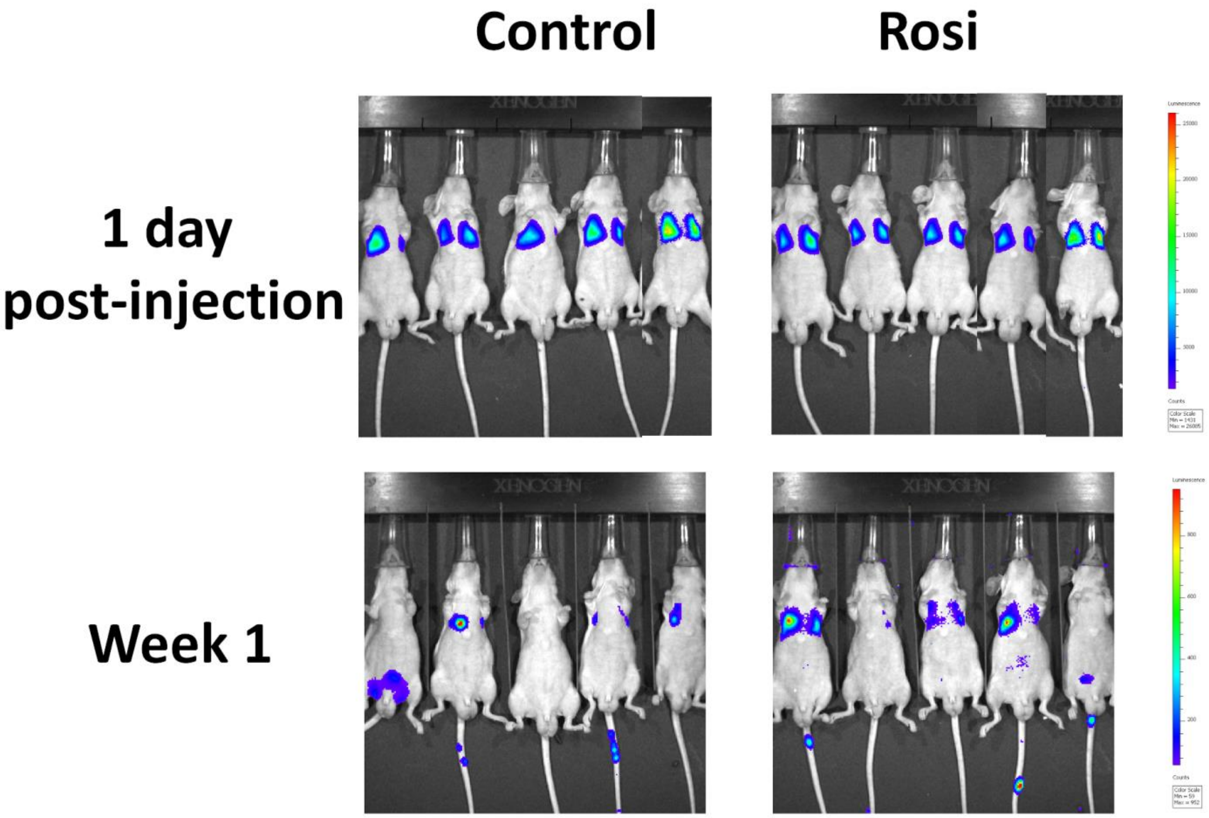

Supplementary Figure SI9 - Rosi decreases migration of tumor cells after tail vein injection

hOS-Saos2-LM7 cells expressing a tk-Luciferase were injected through the tail vein of NOD/SCID mice. Tumor cell dissemination was followed using IVIS imaging. Images of the mice at the time of, and 1 week after injection are shown.
